# Supplementary material for: Forest fragmentation impacts the seasonality of Amazonian evergreen canopies
Source: Nat Commun. 2022 Feb 17;13:917. doi: 10.1038/s41467-022-28490-7 (PMC8854568; doi:10.1038/s41467-022-28490-7)
Supplement: Supplementary file 1 — Supplementary Information [file 41467_2022_28490_MOESM1_ESM.pdf]

Forest fragmentation impacts the seasonality of Amazonian evergreen canopies

AUTHOR LIST

Matheus Henrique Nunes [1, 12], José Luís Campana Camargo [2], Grégoire Vincent [3], Kim Calders [4], Rafael S. Oliveira [5], Alfredo Huete [6], Yhasmin Mendes de Moura [7, 8], Bruce Nelson [9], Marielle N. Smith [10], Scott C. Stark [10], Eduardo Eiji Maeda [1, 11]

AFFILIATIONS

[1] Department of Geosciences and Geography, University of Helsinki, Helsinki, 00014, Finland

[2] Biological Dynamics of Forest Fragment Project, National Institute for Amazonian Research, Manaus, AM, 69067-375 Brazil

[3] AMAP, Univ Montpellier, IRD, CIRAD, CNRS, INRAE, Montpellier, France

[4] CAVElab – Computational and Applied Vegetation Ecology, Department of Environment, Faculty of Bioscience Engineering, Ghent University, Ghent, Belgium

[5] Department of Plant Biology, Institute of Biology, University of Campinas, Campinas, Brazil

[6] School of Life Sciences, Faculty of Science, University of Technology Sydney, Sydney, NSW 2007, Australia

[7] Institute of Geography and Geoecology, Karlsruhe Institute of Technology (KIT), Kaiserstr. 12, 76131, Karlsruhe, Germany

[8] Centre for Landscape and Climate Research, School of Geography, Geology and the Environment, University of Leicester, Leicester, LE17RH, United Kingdom

[9] National Institute of Amazonian Research, Manaus, Brazil

[10] Department of Forestry, Michigan State University, East Lansing, MI, USA

[11] Division of Ecology and Biodiversity, Faculty of Science, The University of Hong Kong

, Hong Kong SAR

[12] Corresponding author (matheus.nunes@helsinki.fi)

SUPPLEMENTARY INFORMATION

Study site and TLS data collection

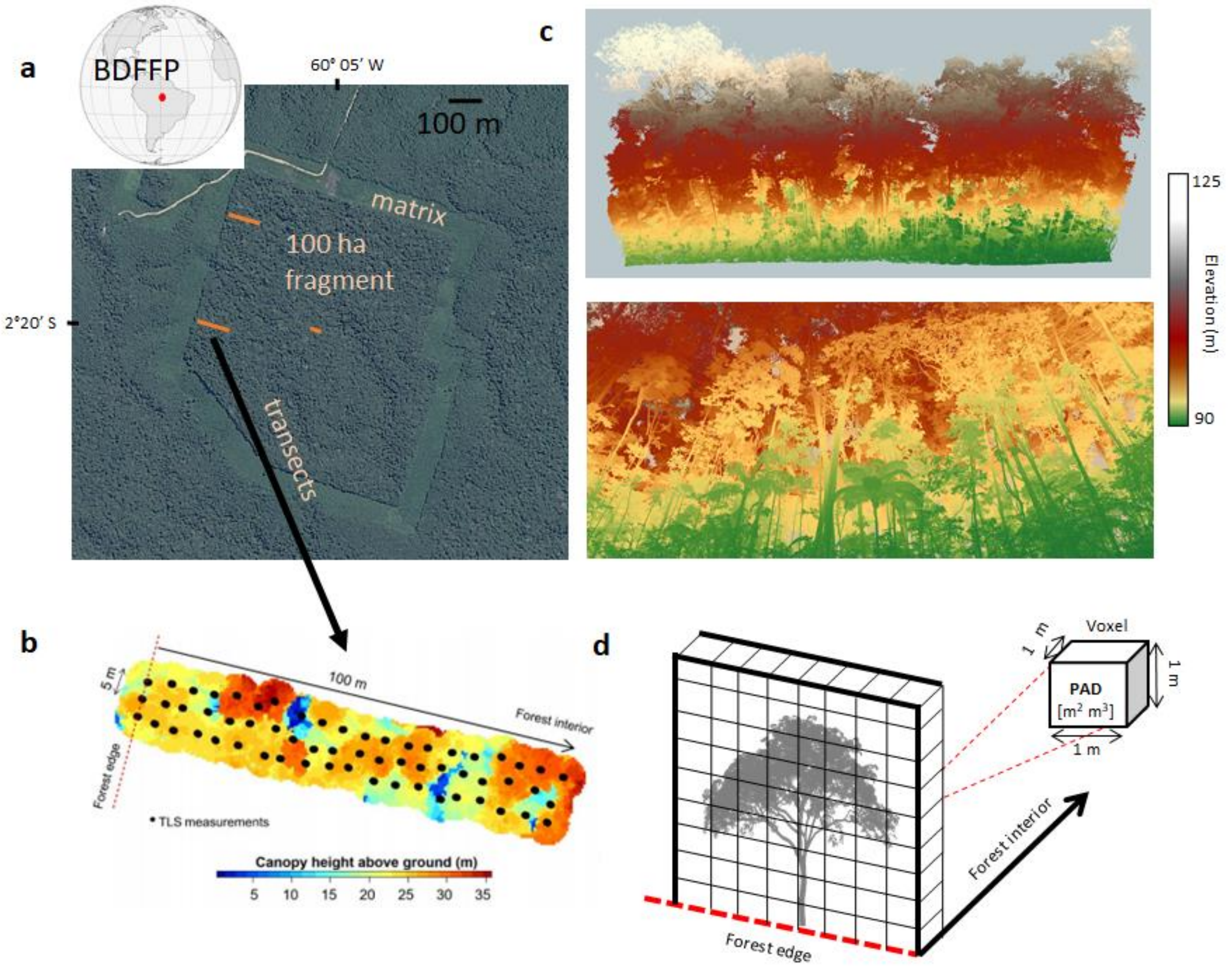

Supplementary Figure 1. Study site and TLS data collection. (a) The Biological Dynamics of Forest Fragments Project (BDFFP), the world's longest-running experimental study of habitat fragmentation, is located in Central Amazonia. The selected 100-ha forest fragment to serve as our experiment is surrounded by a 100 m matrix, regularly cleaned by cutting the regrowth vegetation to keep the forest fragment isolated. Three transects (two of 100 x 10 m at the edges and one of 30 x 10 m at the forest interior) were monitored every two weeks between April and October using a terrestrial LiDAR. (b) Each transect consisted of three scan lines parallel to each other with scans spaced by 5 m within and between lines. Given that the RIEGL VZ-400i has a zenith angle range of 30–130°, an additional scan was acquired at each sampling location with the scanner tilted at 90° from the vertical position. (c) A total of 276 scans across all transects resulted in a complete sampling of the full hemisphere in each scan location. All scans were later co-registered into a single point cloud per

transect. The figures depict a section of a transect's point cloud from a lateral view and from below canopy. (d) Plant area density (PAD, $m^2\ m^{-3}$) for all transects were then calculated using a voxel-based approach (with a 5 m buffer around each transect to maximise the PAD data). The volume occupied by vegetation within each transect was divided into 1 m^3 voxels, and the PAD calculated for each of these voxels.

Edge effects on canopy structure

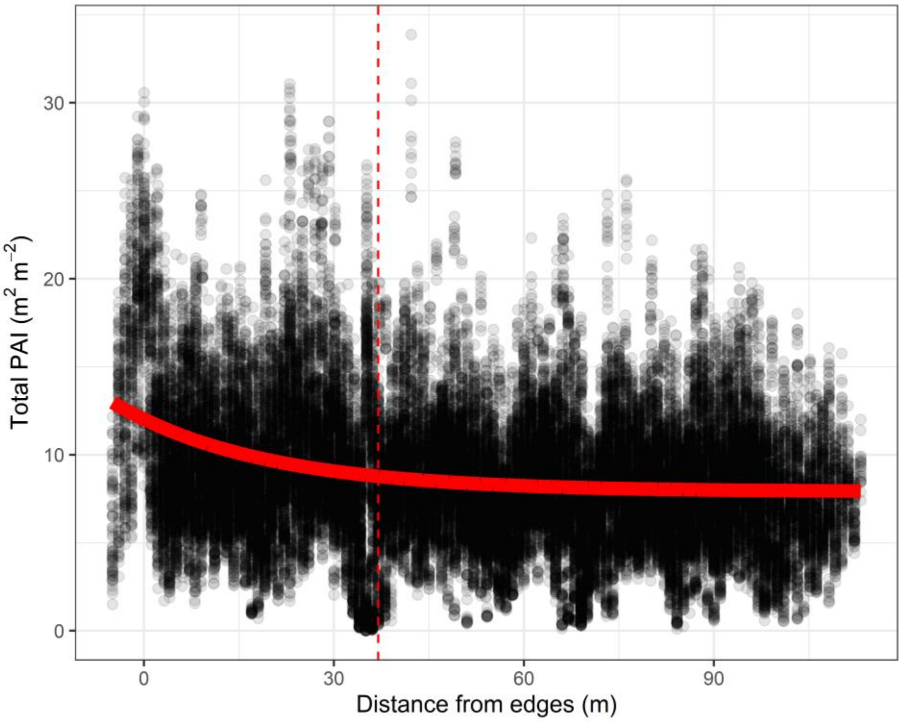

Supplementary Figure 2. Edge effects on canopy structure. Predicted effects of distance from edge (metres) on plant area index (PAI, m^2 $m\text{-}^2$), obtained by fitting non-linear mixed models. The solid red curve is the prediction based on parameter values. The dashed vertical red line corresponds to the optimal edge distance threshold, identified here as 37 m, fitted from a hockey-stick model.

Vertical PAD variation with distance from edges and vertical strata

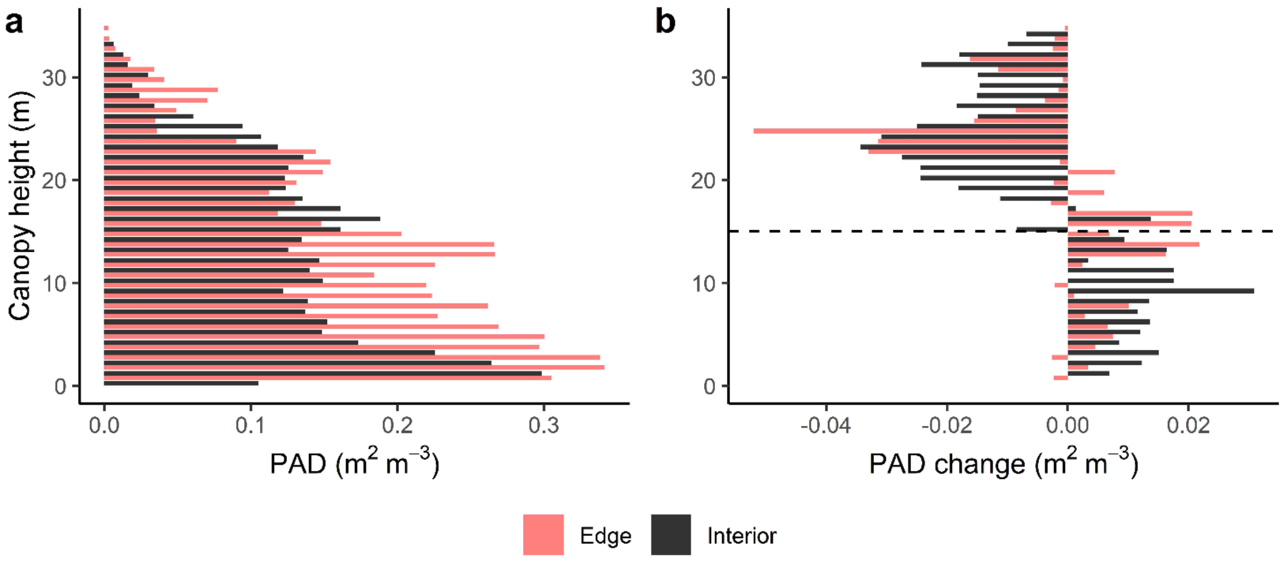

Supplementary Figure 3. Vertical PAD variation with distance from edges and vertical strata. (a) Mean plant area density (PAD) of forest edges (red) and undisturbed forests in the forest fragment interior (black) per 1-m canopy height. (b) PAD changes during the dry season ($PAD_{16th\ October}$ - $PAD_{24th\ June}$) per 1-m canopy height. The dashed horizontal line in panel b represents a canopy height threshold of 15 m indicating a shift in PAD change, which was used as an indication of the variability in forest phenology across the vertical strata (understory versus upper canopy).

Within-transect phenological patterns

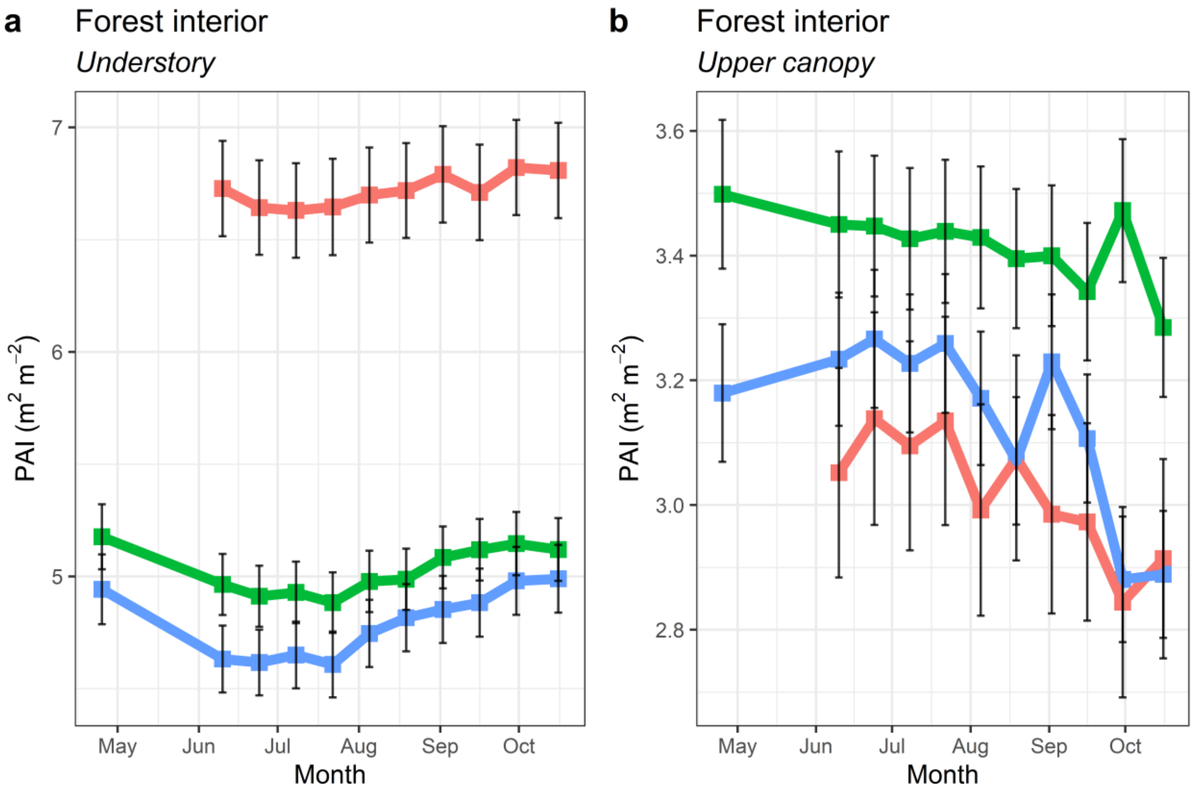

Supplementary Figure 4. Within-transect phenological patterns. Observed Plant Area Index (PAI, m^2 m^{-2}) time-series per transect in interior forests (at least 40 m away from forest edges) in Central Amazonia. Large spatial variation in PAI may be attributed to varying local environmental conditions and species composition. While transects (represented by colour) exhibited similar (a) understory and (b) upper canopy phenological trends, PAI differed significantly between them. Each point in (a) and (b) denotes the mean PAI value per transect (n = 749 for transect orange, 1208 for transect green, 1523 for transect blue) in each measurement time (with lines denoting linear interpolations between points and colours representing the transect identity). Error bars are the 95% confidence intervals.

Distribution of predicted PAI

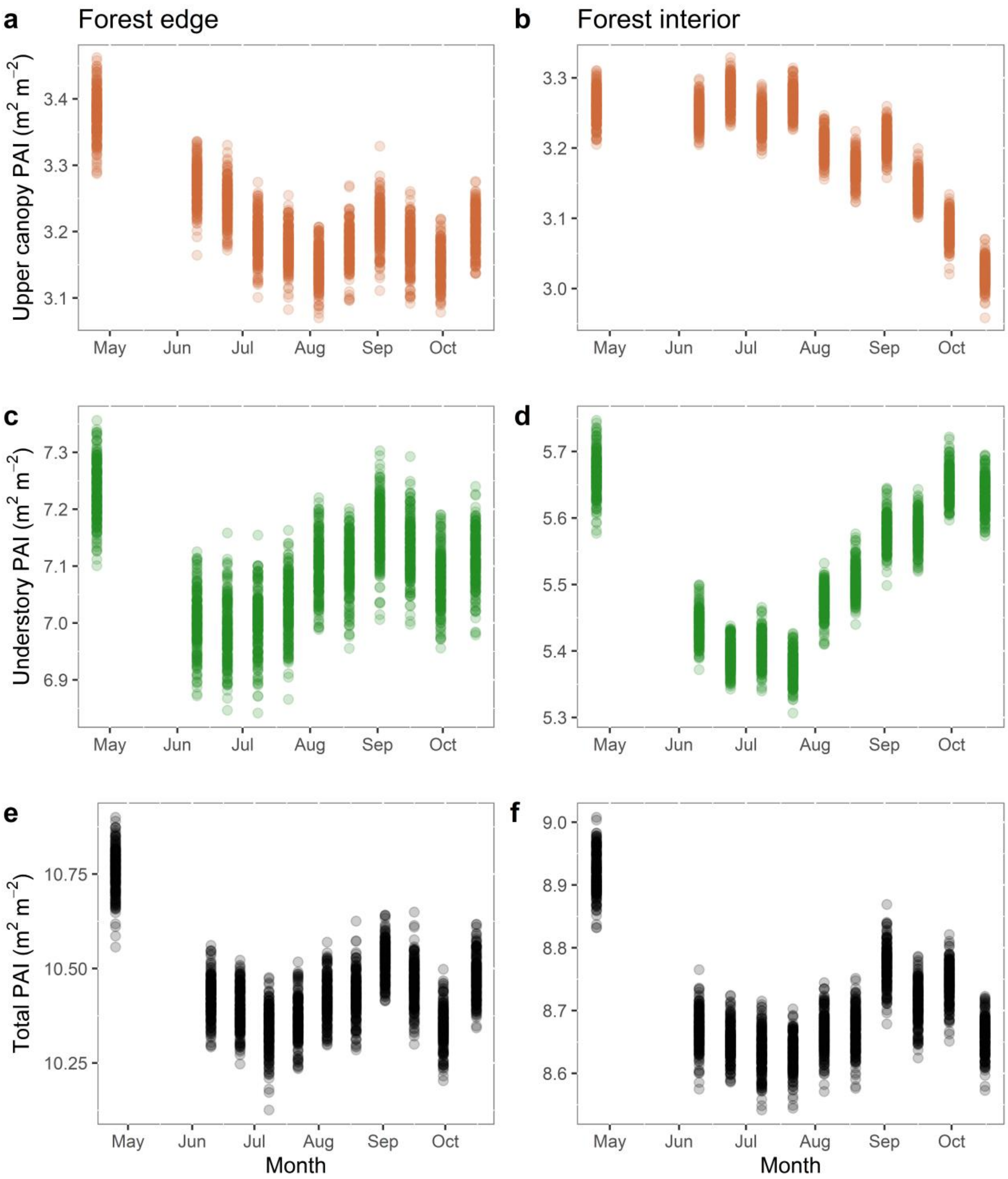

Supplementary Figure 5. Distribution of predicted PAI. PAI predictions from linear mixed modelling used date of LiDAR measurements and the interaction with a categorical variable indicating whether plots were near an edge as fixed variables. Edge effects nested within transect identity were included as random variables. Predicted PAI of the upper canopy (≥ 15 m canopy height, in orange points), the understory (≤ 15 m canopy height, green points), and total PAI that combined

both vertical strata (black points) in forest edges (panels a, c and e) and undisturbed interior forests (panels b, d and f). Forest edges are defined as canopies within 40 m from forest margins while forest interior are canopies at least 40 m away from the forest fragment margins. Each point represents a predicted value; a distribution of predicted points per time was obtained by fitting 200 randomised permutations of subsets split into 80/20 for calibration and validation, respectively.

Upper canopy PAI variation with microclimate data

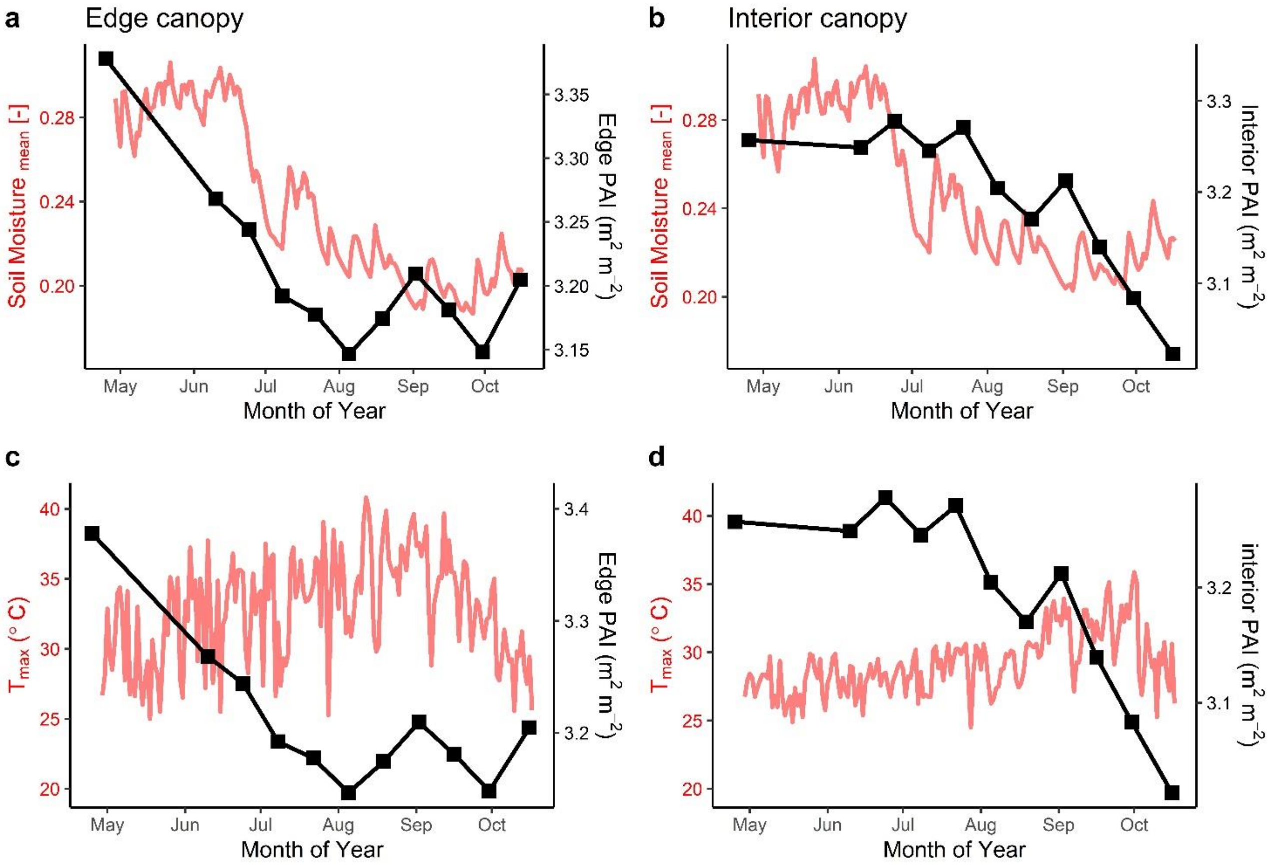

Supplementary Figure 6. Upper canopy PAI variation with microclimate data. Mean daily soil moisture of (a) forest edges and (b) in the interior of forest fragments. Maximum daily temperatures of (c) forest edges and (d) in the interior of forest fragments. Microclimate measurements were continuously obtained every 15 minutes. These microclimatic variables were plotted against the predicted Plant Area Index (PAI) of the upper canopy (> 15 m height) of forest edges and forest interior. Each black point represents the predicted mean PAI values. Red lines correspond to the observed microclimate variables.

Understory PAI variation with microclimate data

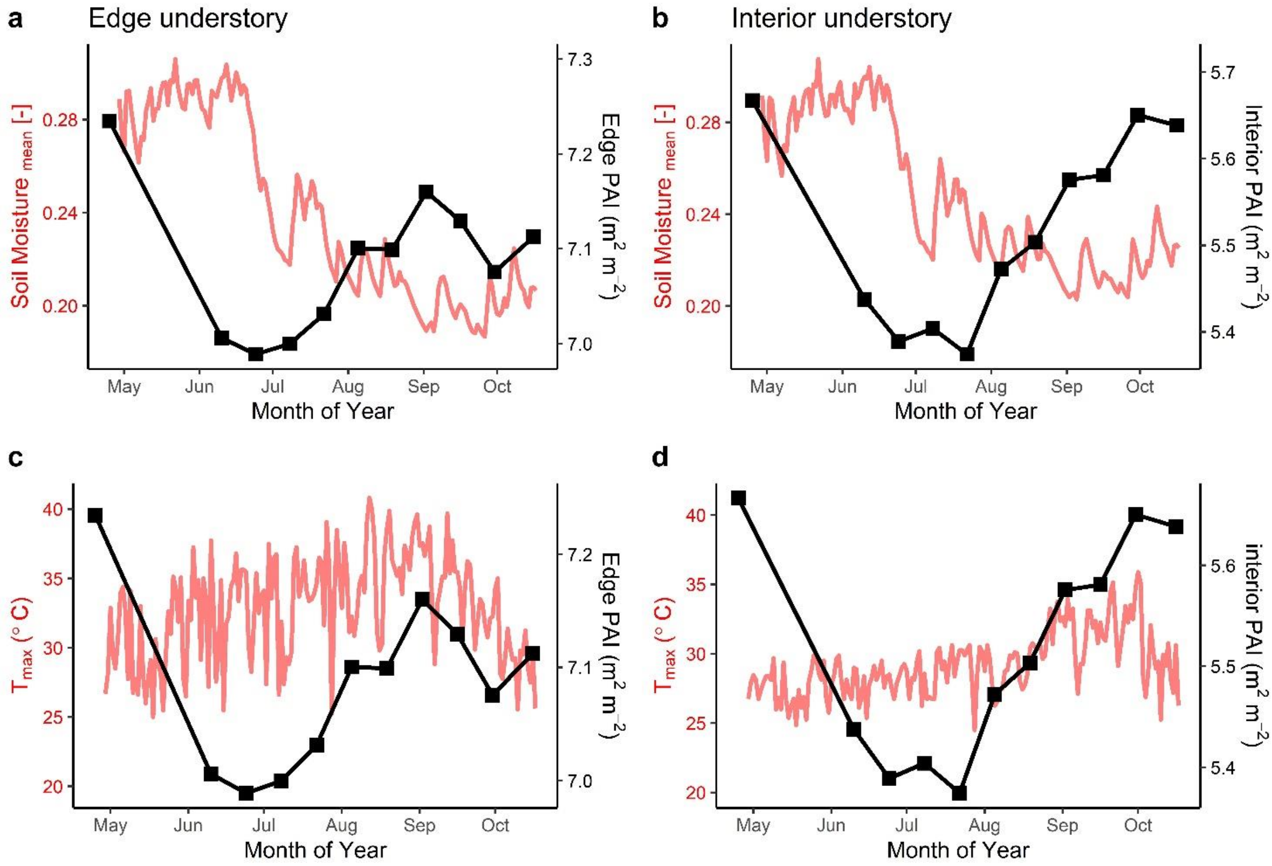

Supplementary Figure 7. Understory PAI variation with microclimate data. Mean daily soil moisture of (a) forest edges and (b) in the interior of forest fragments. Maximum daily temperatures of (c) forest edges and (d) in the interior of forest fragments. Microclimate measurements in the understory of these forests were continuously measured every 15 minutes. These microclimatic variables were plotted against the predicted Plant Area Index (PAI) of the understory (< 15 m height) of forest edges and forest interior. Each black point represents the predicted mean PAI values. Red lines correspond to the observed microclimate variables.

Relationship between the upper canopy and understory PAI

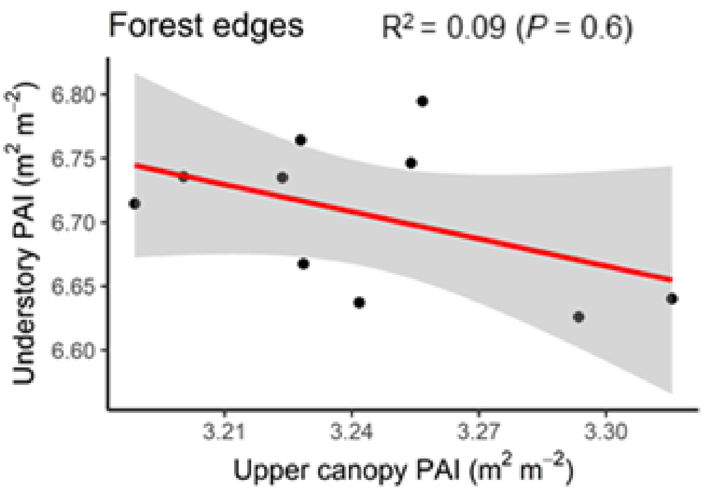

Supplementary Figure 8. Relationship between the upper canopy and understory PAI. Observed seasonal changes in upper canopy LiDAR-based Plant Area Index (PAI, m^2 m^{-2}) versus understory PAI (m^2 m^{-2}) for forest edges without considering the PAI measurements made in April 2019. Black dots represent the mean from 1653 understory and 1653 upper canopy PAI values measured in each survey in the forest edges. Model's R^2 and *P*-value were calculated from simple linear regression (Understory PAI $= \beta_0 + \beta_1$ Upper canopy PAI). The red line in panel b represents predicted values by the model, with the shaded grey area corresponding to the two-sided 95% confidence intervals.

Supplementary Table 1. Model specification and comparison. Results from mixed-effects models (PAI ~ time x edge effects + 1|Transect/Edge effects). We compared this LME model with other LME models that contained the variables time and edge effects to examine the effects of seasonality and fragmentation on PAI variation. Model explanatory power was assessed in terms of AIC. The model with the lowest AIC for each stratum was selected (in bold).

**Model**	**Stratum**	AIC
PAI ~ time + edge effects	Understory	172180.5
PAI ~ time x edge effects	Understory	172184.7
PAI ~ edge effects + time x edge effects	Understory	172192.7
PAI ~ time + time x edge effects	Understory	172162.8
**PAI ~ edge effects + time + time x edge effects**	**Understory**	**172132.4**
PAI ~ time + edge effects	Upper canopy	147854.3
PAI ~ time x edge effects	Upper canopy	147864.9
**PAI ~ edge effects + time x edge effects**	**Upper canopy**	**147843.8**
PAI ~ time + time x edge effects	Upper canopy	147869.4
PAI ~ edge effects + time + time x edge effects	Upper canopy	147889.4
PAI ~ time + edge effects	Total	184706.3
PAI ~ time x edge effects	Total	184683.8
PAI ~ edge effects + time x edge effects	Total	184786.1
PAI ~ time + time x edge effects	Total	184701.3
**PAI ~ edge effects + time + time x edge effects**	**Total**	**184659.4**
