# Supplementary material for: Forest fragmentation impacts the seasonality of Amazonian evergreen canopies
Source: Nat Commun. 2022 Feb 17;13:917. doi: 10.1038/s41467-022-28490-7 (PMC8854568; doi:10.1038/s41467-022-28490-7)
Supplement: Supplementary file 3 — Reporting Summary [file 41467_2022_28490_MOESM3_ESM.pdf]

nature portfolio

Corresponding author(s): Matheus Henrique Nunes
Last updated by author(s): Jan 18, 2022

Reporting Summary

Nature Portfolio wishes to improve the reproducibility of the work that we publish. This form provides structure for consistency and transparency in reporting. For further information on Nature Portfolio policies, see our Editorial Policies and the Editorial Policy Checklist.

Statistics

For all statistical analyses, confirm that the following items are present in the figure legend, table legend, main text, or Methods section.

n/a	Confirmed	
☐	☒	The exact sample size (n) for each experimental group/condition, given as a discrete number and unit of measurement
☐	☒	A statement on whether measurements were taken from distinct samples or whether the same sample was measured repeatedly
☐	☒	The statistical test(s) used AND whether they are one- or two-sided<br>*Only common tests should be described solely by name; describe more complex techniques in the Methods section.*
☐	☒	A description of all covariates tested
☒	☐	A description of any assumptions or corrections, such as tests of normality and adjustment for multiple comparisons
☐	☒	A full description of the statistical parameters including central tendency (e.g. means) or other basic estimates (e.g. regression coefficient) AND variation (e.g. standard deviation) or associated estimates of uncertainty (e.g. confidence intervals)
☐	☒	For null hypothesis testing, the test statistic (e.g. F, t, r) with confidence intervals, effect sizes, degrees of freedom and P value noted<br>*Give P values as exact values whenever suitable.*
☒	☐	For Bayesian analysis, information on the choice of priors and Markov chain Monte Carlo settings
☒	☐	For hierarchical and complex designs, identification of the appropriate level for tests and full reporting of outcomes
☒	☐	Estimates of effect sizes (e.g. Cohen's d, Pearson's r), indicating how they were calculated

Our web collection on statistics for biologists contains articles on many of the points above.

Software and code

Policy information about availability of computer code

Data collection	Repeated Plant Area Index of 1-m3 voxels were collected using a terrestrial laser scanner (TLS) RIEGL VZ-400i<br>Continuous air temperature and soil moisture were measured using TOMST TMS-4 dataloggers
Data analysis	RiSCAN PRO software version 2.9 provided by RIEGL was used to co-register all the data points collected using the TLS;<br>Inverse distance weighting algorithm: LASTools (rapidlasso, GmbH, Germany);<br>PAD calculation: AMAPVox (CIRAD, Institut de Recherche pour le Développement, France);<br>Temperature and volumetric moisture: TMS Lolly manager (provided by Tomst, Czech Republic);<br>The following packages in the Software R Core Team (2018) were used for all the statistical analysis: lidR ; nlme ; SiZer ;

For manuscripts utilizing custom algorithms or software that are central to the research but not yet described in published literature, software must be made available to editors and reviewers. We strongly encourage code deposition in a community repository (e.g. GitHub). See the Nature Portfolio guidelines for submitting code & software for further information.

Data

Policy information about availability of data

All manuscripts must include a data availability statement. This statement should provide the following information, where applicable:

- Accession codes, unique identifiers, or web links for publicly available datasets
- A description of any restrictions on data availability
- For clinical datasets or third party data, please ensure that the statement adheres to our policy

Repeated Plant Area Index (PAI) data collected using a terrestrial laser scanner (TLS) between April and October 2019 in Central Amazonia have been deposited in the national Finnish Fairdata services and were made publicly available with the identifier https://etsin.fairdata.fi/dataset/e488f81b-b927-4bbd-a6f7-2f532f434b2b. Microclimate data collected in the field and estimated solar radiation and rainfall data analysed during the current study are available from the corresponding author on reasonable request.

Field-specific reporting

Please select the one below that is the best fit for your research. If you are not sure, read the appropriate sections before making your selection.

☐ Life sciences ☐ Behavioural & social sciences ☒ Ecological, evolutionary & environmental sciences

For a reference copy of the document with all sections, see nature.com/documents/nr-reporting-summary-flat.pdf

Ecological, evolutionary & environmental sciences study design

All studies must disclose on these points even when the disclosure is negative.

Study description	We investigated the phenology of forests in Central Amazonia with terrestrial laser scanning (TLS, also terrestrial LiDAR) surveys collected every 15 days spanning the wet and dry seasons. We combined the TLS measurements with microclimate data to investigate how forest fragmentation and microclimatic seasonality interact to affect plant area of the understory and the upper canopy.
Research sample	The study was conducted in Central Amazonian forests (2°20 30 'S, 60° 05 37 W) within the Biological Dynamics of Forest Fragments Project (BDFFP), the world's longest-running experimental study of habitat fragmentation. The region has seen notable carbon and biodiversity losses due to forest fragmentation effects and is predicted to be markedly impacted by climatic changes. The pioneering BDFFP project sites are composed of forest fragments originally isolated in 1980 by converting mature forest into cattle pastures. Currently, the matrix is dominated by secondary growth forests, but a 100 m strip surrounding the forest fragments is regularly cleaned by cutting vegetation regrowth to keep the forest fragments isolated. As an experimental control that minimises anthropogenic influences as confounding factors, such as illegal logging, hunting, fire penetration and pollution, the project offers unique insights into ecological and environmental changes in fragmented forests. We selected a 100-ha forest fragment to investigate phenological responses with varying distances from the fragment edges (0 – 500 m). At the community level, the forest edges of our study are dominated by a high density of early-successional, fast-growth species, because of the elevated tree mortality near forest edges and seed dispersion from degraded neighbouring habitats, while the centre of the fragment comprises of undisturbed primary forests.
Sampling strategy	The TLS scans covered two transects of 100 x 10 m perpendicular to the forest fragment margins measured 11 times and 1 transect of 30 x 10 m length in the centre of the forest fragment measured 10 times. The transect in the centre lies 500 m from any fragment margin to ensure sampling of forest interior and that effectively there were no edge effects. This sampling strategy resulted in 276 scans across all transects each time, with a complete sampling of the full hemisphere in each scan location. This sampling strategy covered a total area of 0.52 ha, which included 274 trees with diameter at breast height (DBH) > 10 cm, lianas, shrubs, saplings, seedlings and acaulescent palms that were repeatedly measured 11 times. To ensure a full 3D representation of the upper canopy, each transect consisted of three scan lines parallel to each other with scans spaced at 5 m intervals within and between lines. The distance between scanning positions was smaller than the 10-40 m usually applied in previous studies to minimize data uncertainties due to occlusion in dense tropical forests and maximize data acquisition in the upper canopy. Given that the RIEGL VZ-400i has a zenith angle range of 30–130°, an additional scan was acquired at each sampling location with the scanner tilted at 90° from the vertical position.<br><br>We measured air temperature (°C) and electrical conductivity of soil moisture (time-domain transmission; TDT) across a network of 22 data loggers every 15 minutes varying in distance from the forest fragment margins (0 and 520 m).
Data collection	TLS-based PAI data were acquired using a RIEGL VZ-400i system between April and October 2019 every 15 days, except between the end of April and early June when the time difference between measurements was 40 days (we clarify in the analysis section how we addressed artefacts attributed to sampling effort). Matheus H Nunes led the field campaigns.<br><br>We derived solar radiation from the product MCD18A2 V6 (https://lpdaac.usgs.gov/products/mcd18a2v006/).<br><br>Meteorological parameters are derived from the NASA's GMAO MERRA-2 assimilation model (https://gmao.gsfc.nasa.gov/reanalysis/MERRA/) and GEOS FP-IT (https://gmao.gsfc.nasa.gov/news/geos_system_news/2016/FP-IT_NRT_G5.12.4.php).

	Microclimate measurements: Data loggers were shielded from direct solar radiation and recorded data every 15 minutes. Microclimate data were recorded between 27th April 2019 and 16th October 2019, resulting in a total of 435,798 coupled temperature and volumetric soil moisture readings.
Timing and spatial scale	TLS data in each scan position (out of 276 scan positions) started on 25th April 2019 and were acquired every 15 days until 16th October 2019. The total sampling area covered an area of 0.52 ha monitored 11 times during the above-mentioned time period. We observed consistent positive PAD changes below the height of 15 m above the ground and negative PAD changes above 15 m height. Thus, given the existence of only two axes of variation along the vertical profile of the vegetation, we utilized this height to define understory (< 15 m aboveground) and upper canopy (> 15 m aboveground) in this study. The sum of all the understory PADs and the upper canopy PADs are referred to as understory PAI and upper canopy PAI, respectively. Our analysis comprises of 5,133 PAI values for the understory and 5,133 PAI values for the upper canopy, each monitored 11 times during the seasonal climatic variations.
Data exclusions	No data were excluded from the analysis
Reproducibility	Our experiment did not depend on reproducibility - the results from each measurement were taken into account and used as input data for our prediction models.
Randomization	Transects were treated as random factors in the mixed modelling. Transect identity was included as a random effect, allowing us to include any idiosyncratic differences between transects, with a random intercept term capturing variation in PAI between transects. The LME model was fitted using the lme function in the nlme R package.
Blinding	Blinding is not relevant to our study. No persons were used in the study.

Did the study involve field work? ☒ Yes ☐ No

Field work, collection and transport

Field conditions	Canopy measurements were made between April and October 2019. Field conditions varied enormously though seasons as stated in Figure 2 of the main text.
Location	The study is located in Central Amazonia, Dimona experimental area) within the Biological Dynamics of Forest Fragments Project (2° 20 30 'S, 60° 05 37 W).
Access & import/export	Permit to conduct research in the area was granted by ICMBio (Instituto Chico Mendes da Biodiversidade). We did not import or export any material.
Disturbance	No disturbance was caused by this study.

Reporting for specific materials, systems and methods

We require information from authors about some types of materials, experimental systems and methods used in many studies. Here, indicate whether each material, system or method listed is relevant to your study. If you are not sure if a list item applies to your research, read the appropriate section before selecting a response.

Materials & experimental systems

n/a	Involved in the study	
☒	☐	Antibodies
☒	☐	Eukaryotic cell lines
☒	☐	Palaeontology and archaeology
☒	☐	Animals and other organisms
☒	☐	Human research participants
☒	☐	Clinical data
☒	☐	Dual use research of concern

Methods

n/a	Involved in the study	
☒	☐	ChIP-seq
☒	☐	Flow cytometry
☒	☐	MRI-based neuroimaging
